# Geographical distribution and driving force of microbial communities in the sediments of Diamantina and Kermadec trenches

**DOI:** 10.3389/fmicb.2024.1474645

**Published:** 2024-11-18

**Authors:** Yue Zhang, Hongmei Jing, Hao Liu

**Affiliations:** ^1^CAS Key Lab for Experimental Study under Deep-Sea Extreme Conditions, Institute of Deep-Sea Science and Engineering, Chinese Academy of Sciences, Sanya, China; ^2^HKUST-CAS Sanya Joint Laboratory of Marine Science Research, Chinese Academy of Sciences, Sanya, China

**Keywords:** driving force, biogeographical distribution, bacteria, micro-eukaryotes, hadal trenches

## Abstract

The distinctive geological characteristics of hadal trenches are recognized to affect the construction and ecological role of microbial communities; however, information on their population dynamics and assembly processes remains limited. In this study, bacteria and micro-eukaryotes in the sediments of the Diamantina and Kermadec trenches were explored utilizing high-throughput sequencing. Compared to the Diamantina Trench, significantly lower levels of bacterial and micro-eukaryotic biodiversity (*p* < 0.01), bacterial gene copy number (*p* < 0.05), and heterotrophic/parasitic micro-eukaryotic proportions (*p* < 0.05) were detected in the Kermadec Trench, which also exhibited a low community complexity based on the network analysis. Within each trench, no obvious population shifts were observed along the trench axis. Microbial communities in both trenches showed clear distance–decay distributions, mainly driven by stochastic processes. This study provided fresh perspectives on the microbial community assembly mechanism in deep-sea trenches. Studies of community complexity and diversified trophic states of microbes would contribute to an improved understanding of ecological functions and diversification in this extreme biosphere.

## 1 Introduction

The deep sea differs from other environments by nearly complete darkness, a relatively low average temperature, high hydrostatic pressure, and a limited amount of organic matter (Jørgensen and Boetius, 2007). Marine microbiomes (primarily consisting of bacteria, archaea, micro-eukaryotes, and viruses) perform crucial functions in the marine food webs and global biogeochemical cycles (Sunagawa et al., 2015). Deep-sea microbes possess a high degree of adaptability to the harsh conditions of their environment, leading to the evolution of distinctive biodiversity and metabolic mechanisms (Wang et al., 2013). For example, mixotrophic and chemoautotrophic microbial processes supply extra organic carbon sources and jointly modulate any imbalance that emerges between the vertical flux input of organic carbon and its biological utilization (Reinthaler et al., 2010). Systematic investigation of the distribution, composition, and interactions of deep-sea microbiomes will enhance our comprehension of the structure and ecological functions of deep-ocean ecosystems.

Assembly processes determine microbial biodiversity and community composition, thereby governing its functions (Zhang et al., 2023). Both determinism and stochasticity determine microbial community assembly, and their relative contributions vary with different environments (Machado et al., 2019). Trenches, deepest oceanic areas with isolated hydrotopographical situations and highly elevated hydrostatic pressure (e.g., >60 MPa) (Jamieson et al., 2010), generally support a variety of hadal organisms with a high level of endemism and density (Jamieson et al., 2010). In the Yap Trench sediment, the whole prokaryotic community assembly is driven by stochastic processes; meanwhile, homogeneous selection (32.6–52.9%) belonging to a deterministic process, governs the prokaryotic community assembly in hadal sediments as sediment depth increases (Sun et al., 2024). In the Mariana Trench, deterministic processes governed macrofaunal community assembly in the arc and ridge systems (Giguère and Tunnicliffe, 2021). In contrast, the biogeography of the micro-eukaryotic community in the seamount sediments of the Yap and Mariana trenches was mainly driven by the deterministic process (Zhang et al., 2023) and affected by the interaction with prokaryotes as well. These complex cross-kingdom biotic interactions and niche sharing would help to maintain the diversity and stability of microbial communities (Faust and Raes, 2012). Geological and physicochemical conditions were highly varied within and between prokaryotes and micro-eukaryotes in different hadal trenches; thus, it would be necessary to elucidate the driving forces for microbial communities living in trenches with different spatial scales.

Kermadec is the fourth of five global trenches with a depth of more than 10,000 m. It reaches a maximum depth of 10,047 m and is situated approximately 120 km away from the northeastern coast of New Zealand in the Southern Hemisphere (Angel, 1982). The Diamantina Trench, in fact, a deep seafloor fracture zone (Diamantina Fracture Zone), is situated in the Indian Ocean and its maximum depth is roughly 7,079 m (Stewart and Jamieson, 2019). A prominent enrichment of heterotrophic bacterial populations in the sediments of the Kermadec Trench has been proposed to be highly related to organic matter degradation and recalcitrant material breakdown (Peoples et al., 2019). Comparatively, microbes in the Diamantina Trench were generally unknown, due to its remote location. Considering the substantial variations in microbial cell abundance and community structures across different trenches (Hiraoka et al., 2019; Peoples et al., 2019; Liu et al., 2020), we hypothesize distinct community assembly processes between prokaryotes and micro-eukaryotes in different trenches.

In this study, we collected sediment samples along the axis from the Diamantina and Kermadec trenches and investigated the diversity, composition, and community assembly of bacterial and micro-eukaryotic communities utilizing high-throughput sequencing. This study intended to uncover (i) the geographic variability of bacteria and micro-eukaryotic communities along the axis of each trench, (ii) the heterogeneity of microbial communities between the two trenches, and (iii) the relative importance of diverse driving forces, such as spatial variables, environmental factors, and bio-interactions, for the assembly of bacterial and micro-eukaryotic communities.

## 2 Materials and methods

### 2.1 Sample collection and physicochemical parameter measurement

**Figure 1:**
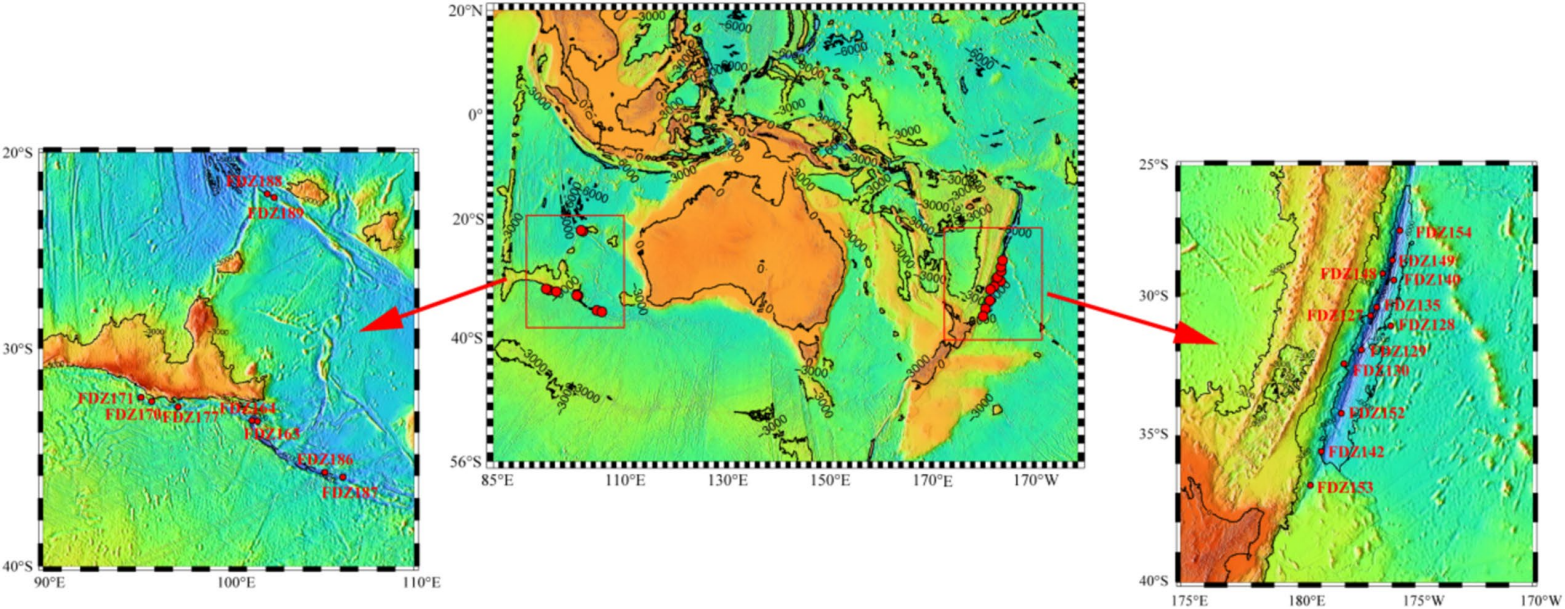
A map showing the sampling locations in the Diamantina and Kermadec trenches.

In total, 21 pushcore sediment samples were obtained from the Diamantina (9 samples) and Kermadec (12 samples) trenches in the cruise TS29 by submersible “Fendouzhe” from November 2022 to March 2023 (Figure 1). The surface layer (0–4 cm) of pushcore sediment was sliced and immediately stored at −80°C. *In situ* hydrographic characteristics (that is, locations and depths) were collected from the full-ocean-depth manned submersible “Fendouzhe.” Approximately 5 g of frozen sediment was analyzed for sediment properties, such as total organic carbon (TOC), total nitrogen (TN), nitrate (NO_3_^−^), and ammonia (NH_4_^+^), as described previously (Wang et al., 2016). Briefly, the sediments were oven-dried at 105°C and then measured with an elementary analyzer (vario MACRO cube, Germany) to determine the concentrations of TOC and TN. NO_3_^−^and NH_4_^+^ were determined after being processed with 1 M HCl, followed by analysis using a Seal Analytical AA3 continuous flow autoanalyzer.

### 2.2 DNA extraction, polymerase chain reaction (PCR) amplification, and sequencing

The total DNAs were obtained from the surface layers of trench sediments (~0.5 g per sample) using the MoBio PowerSoil DNA extraction kit following the manufacturer’s instructions. The concentrations of acquired DNAs were measured using the fluorometer (Qubit 2.0 Life Technologies), and the quality was assessed using gel electrophoresis. For each genomic DNA sample, independent triplicates were extracted and pooled together (~250 mg) as templates for PCR amplification. The V3–V4 region of 16S rRNA and the V4 region of 18S rRNA genes were then amplified with Bac338F (5′-TCCTACGGGAGGCAGCAGT-3′) and Bac806R (5′-GGACTACCAGGGTATCTAATCCTGTT-3′) (Oppliger et al., 2008), as well as TAReuk454FWD1 (5′-CCAGCA(G/C)C(C/T)GCGGTAATTCC-3′) and REV3 (5′-ACTTTCGTTCTTGAT(C/T)(A/G)A-3′) (Stoeck et al., 2010), respectively. The PCR system and reaction were performed as previously described (Zhang et al., 2023; Zhang and Jing, 2024). A negative control of double-distilled water was utilized to avoid reagent contamination. The amplicons were sequenced using an Illumina HiSeq PE250 platform (Novogene Co., Ltd.).^1^

### 2.3 Quantitative PCR

The gene abundance of bacterial 16S rRNA and micro-eukaryotic 18S rRNA was quantified via a StepOnePlus Real-Time PCR system (Bio-Rad Laboratories) with Bac338F (5′-TCCTACGGGAGGCAGCAGT-3′) and Bac806R (5′-GGACTACCAGGGTATCTAATCCTGTT-3′), as well as EUK345F (5′-AAGGAAGGCAGCAGGCG-3′) and EUK499R (5′-CACCAGACTTGCCCTCYAAT-3′), respectively. The system, reactions, and calibrations of qPCR were conducted according to a protocol that was previously reported (Zhang et al., 2023; Zhang and Jing, 2024). For each sample, triplicate qPCR reactions were carried out, with the efficiencies being 101.7 and 91.8%, respectively, and then, copy numbers of genes were normalized in terms of the number of sequences.

### 2.4 Bioinformatics analysis

Raw paired-end sequences were trimmed, merged, denoised, and filtered for chimera with the DADA2 (version 1.16, Callahan et al., 2016) plug-in of QIIME 2 (version 2023.2, Caporaso et al., 2010), and the representative sequences according to 100% similarity were picked out. Taxonomic and compositional analyses of 16S rRNA and 18S rRNA sequences were performed against SILVA (version 138) and PR^2^ databases (Guillou et al., 2013) for bacteria and micro-eukaryotes, respectively. After removing singletons, amplicon sequence variants (ASVs) identified as chloroplasts, mitochondria, or eukaryotes for bacteria sequences and ASVs that were not related to micro-eukaryotes (including archaea, bacteria, metazoan, and plastidial sequences) were excluded as well. A filtered ASV table for each sample was produced using QIIME 2, based on the Shannon diversity index.

The community composition and sequence abundance were presented with the “ggplot2” packages in R (version 4.3.3). Venn diagrams were displayed for the indigenous and shared ASVs with the “vegan” package (Oksanen et al., 2018). For bacteria, potential carbon, nitrogen, and sulfur cycle-related pathways were predicted by Tax4Fun based on the SILVA (version 138) database (Asshauer et al., 2015), and the results were presented via bubble plots with “ggplot2.” For micro-eukaryotes, ASVs were categorized into trophic groups with the highest level of available information, as described previously (Zhang et al., 2023). The distribution and composition of trophic status between the two trenches were performed by a stacking diagram in R (version 4.3.3).

### 2.5 Statistical analysis

Based on the Bray–Curtis resemblance matrix, the non-metric multidimensional scaling (nMDS) and an analysis of similarities (ANOSIM) were applied to evaluate community similarity and significant differences among different sampling sites with Paleontological Statistics (PAST, version 3) (Hammer et al., 2001). According to the relative abundance of each ASV, the significant differences between the two trenches were calculated by two-sided Welch’s t-test with Benjamin–Hochberg False Discovery Rate (FDR) correction using the Statistical Analysis of Metagenomic Profiles (STAMP, version 2.1.3, Parks et al., 2014), and the results were displayed using extended error bar plots.

The distance–decay relationship in the microbial communities was determined using a linear least-squares regression between community dissimilarity and geographic distance with the “stats” package. The null model analysis (Dini-Andreote et al., 2015) was conducted to assess the relative contribution of ecological processes in controlling the beta diversity of bacterial and micro-eukaryotic communities. In short, the mean phylogenetic distances between the two closest ASVs were determined using the abundance-weighted beta-mean nearest taxon distance (βMNTD). The beta-nearest taxon index (βNTI) was calculated as previously reported (Dini-Andreote et al., 2015). A significant deviation (that is, |βNTI| > 2) means the dominance of selection and deterministic processes, whereas a low deviation (that is, |βNTI| < 2) indicates the dominance of stochastic ecological processes, such as dispersal and drift (Stegen et al., 2013).

According to the longest gradient lengths of preliminary detrended correspondence analysis (DCA), Canonical correspondence analysis (CCA) or redundancy analysis (RDA) was used to analyze the environmental effects on bacterial and micro-eukaryotic communities with CANOCO software (version 5.0, Šmilauer and Lepš, 2014). Analysis of networks indicating the co-occurrence modules within/between various taxonomic groups was performed based on the 200 most abundant ASVs from respective bacteria and micro-eukaryotes. The correlation matrix, *r*-, and *p*-values were computed using a similarity matrix with the “psych” package (Revelle, 2015). Statistically significant correlations (Spearman’s |*r*| > 0.8 and FDR-adjusted *p* < 0.05) were further presented using Gephi (version 0.9.3, Bastian et al., 2009).

## 3 Results

### 3.1 Hydrographic conditions

The Kermadec Trench is located in the Southern Pacific Ocean and was formed by the subduction of the Pacific plate to the Australian plate. The Diamantina Trench, situated in the Indian Ocean, was shaped by the geological breakup of the Australian and Antarctic continents. Sediment samples along the axis were collected from the abyssal-hadal zone in the Diamantina (5,383–10,100 m) and Kermadec (5,163.8–6,802.3 m) trenches. Comparatively, significantly higher TOC contents (*p* < 0.05) and slightly higher averaged TP and NO_3_^−^ concentrations were detected in the Diamantina Trench, compared to the Kermadec Trench. The highest concentration of TOC was detected at Stn. FDZ188 in the Diamantina Trench (i.e., 17,043.50 mg/kg). As for NH_4_^+^, the highest and lowest concentrations were observed at Stns. FDZ140 and FDZ135 in the Kermadec Trench, respectively.

### 3.2 Community structure and sequence abundance

Regarding the high-quality reads, 608,930 reads and 2,229 ASVs were obtained for the bacteria community, and the highest and lowest numbers of ASVs were, respectively, detected at Stn. FDZ186 in the Diamantina Trench and Stn. FDZ127 in the Kermadec Trench (Table 1). For the micro-eukaryotic community, 901,060 sequences and 3,744 ASVs were produced, and the highest and lowest numbers of ASVs were found at Stn. FDZ188 in the Diamantina Trench and Stn. FDZ129 in the Kermadec Trench, respectively (Table 1).

**Table 1:** Locations and sequence details of surface sediments in the Diamantina and Kermadec trenches.

Regions	Stns.	Lon.	Lat. (°S)	Depth (m)	Original reads (Bac.)	Quality reads (Bac.)	ASVs (Bac.)	Original reads (Euk.)	Quality reads (Euk.)	ASV (Euk.)
Diamantina Trench	FDZ163	101.55°E	33.47	6,802	62,739	19,301	759	53,158	41,015	245
	FDZ164	101.26°E	33.44	6,792	60,459	20,118	841	99,751	84,731	146
	FDZ170	95.85°E	32.54	5,164	82,308	19,628	965	101,534	85,403	189
	FDZ171	95.28°E	32.34	5,322	54,948	13,125	820	60,175	48,474	483
	FDZ177	97.27°E	32.80	5,657	69,184	23,224	388	68,900	51,628	159
	FDZ186	105.19°E	35.83	6,574	61,162	20,511	1,066	59,087	42,442	302
	FDZ187	106.16°E	36.05	6,349	80,114	42,182	859	34,986	2,118	610
	FDZ188	102.45°E	22.37	6,671	61,485	20,941	1,010	–	–	-
	FDZ189	102.08°E	22.17	6,491	60,845	16,662	682	43,594	14,723	831
Kermadec Trench	FDZ127	176.91°W	30.69	7,600	78,505	37,692	140	95,324	65,916	194
	FDZ128	176.05°W	31.07	5,825	71,003	30,076	141	–	–	–
	FDZ129	177.30°W	31.94	10,100	102,073	51,012	148	42,458	6,994	32
	FDZ130	178.04°W	32.45	5,861	55,845	24,367	155	–	–	–
	FDZ135	176.65°W	30.38	9,500	58,862	24,502	173	108,710	107,455	190
	FDZ140	175.92°W	29.37	7,800	71,084	28,387	177	68,540	50,212	243
	FDZ142	179.00°W	35.57	6,471	70,883	28,549	143	50,704	29,952	227
	FDZ148	176.31°W	29.11	6,390	106,336	42,716	271	85,594	52,875	295
	FDZ149	175.97°W	28.62	9,286	89,082	38,816	156	106,407	85,868	153
	FDZ152	178.15°W	34.23	6,969	92,721	38,919	167	55,445	41,866	213
	FDZ153	179.47°W	36.74	5,383	62,486	33,427	350	86,284	62,108	330
	FDZ154	175.66°W	27.50	9,639	61,247	34,775	323	44,482	27,280	156

Bac., bacteria; Euk., micro-eukaryote.

**Figure 2:**
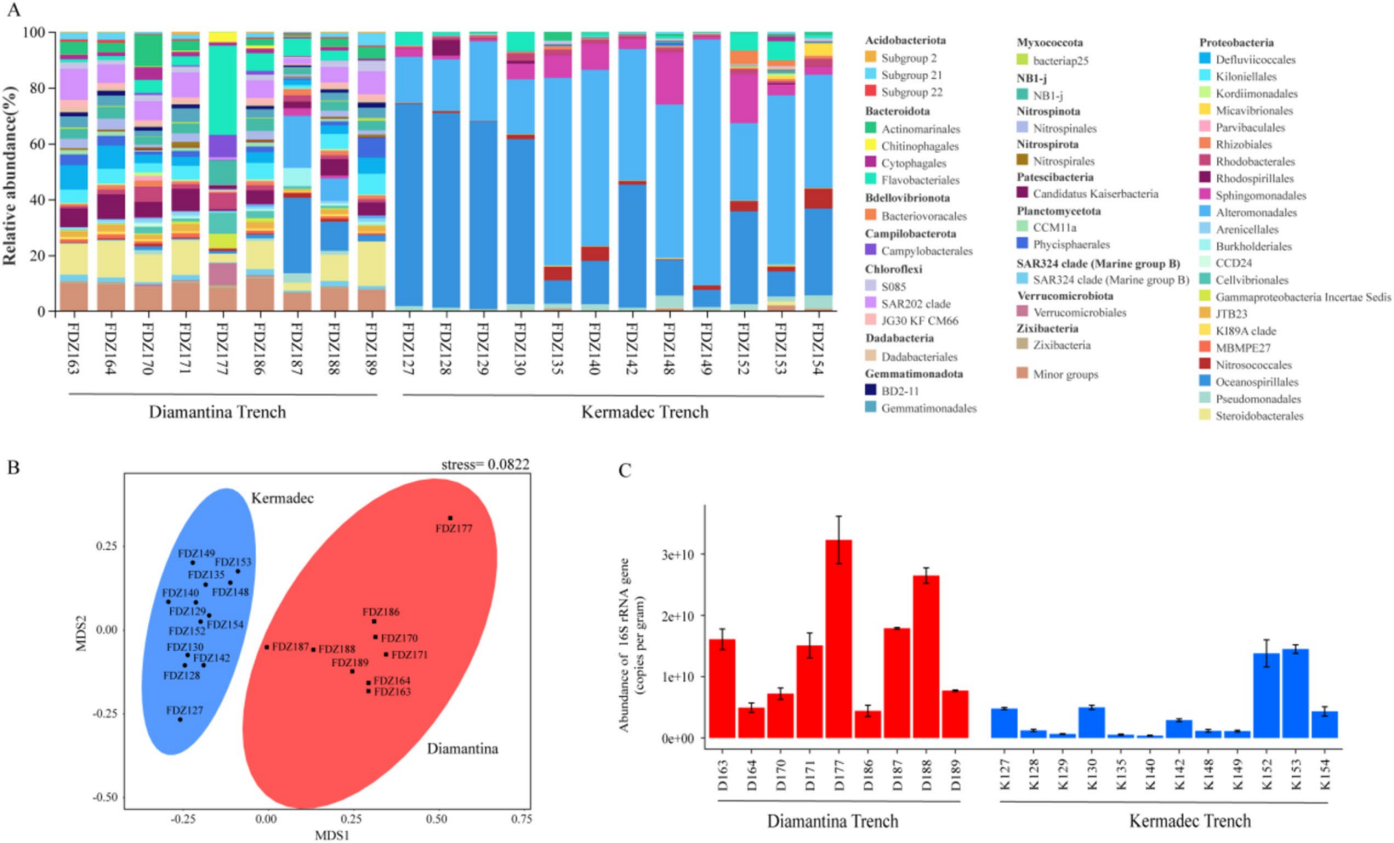
Community structure (A), NMDS plot (B), and gene copy number of 16S rRNA (C) of bacteria in the sediment of the Diamantina and Kermadec trenches. Error bars represent standard deviation.

For the community structure, Actinobacteriota, Chloroflexi, and Proteobacteria were the predominant phyla detected among the bacterial sequences (Figure 2A). Actinomarinales, Steroidobacterales, and SAR202 clade were more abundant in the sequence data from Diamantina Trench, while Gammaproteobacteria Alteromonadales and Oceanospirillales represented a higher percentage of the sequence dataset in the Kermadec Trench. Along the axis of the trench, the percentage of Oceanospirillales was significantly higher in the middle than that in the northeast and southwest ends (*p* < 0.05) and had the opposite trend with Alteromonadales. NMDS analysis revealed that bacterial community structures from the two trenches were significantly different (ANOSIM, *p* < 0.01) (Figure 2B). Comparatively, significantly lower 16S rRNA sequence abundance was observed in the Diamantina Trench than that in the Kermadec Trench (ANOSIM, *p* < 0.05) (Figure 2C).

**Figure 3:**
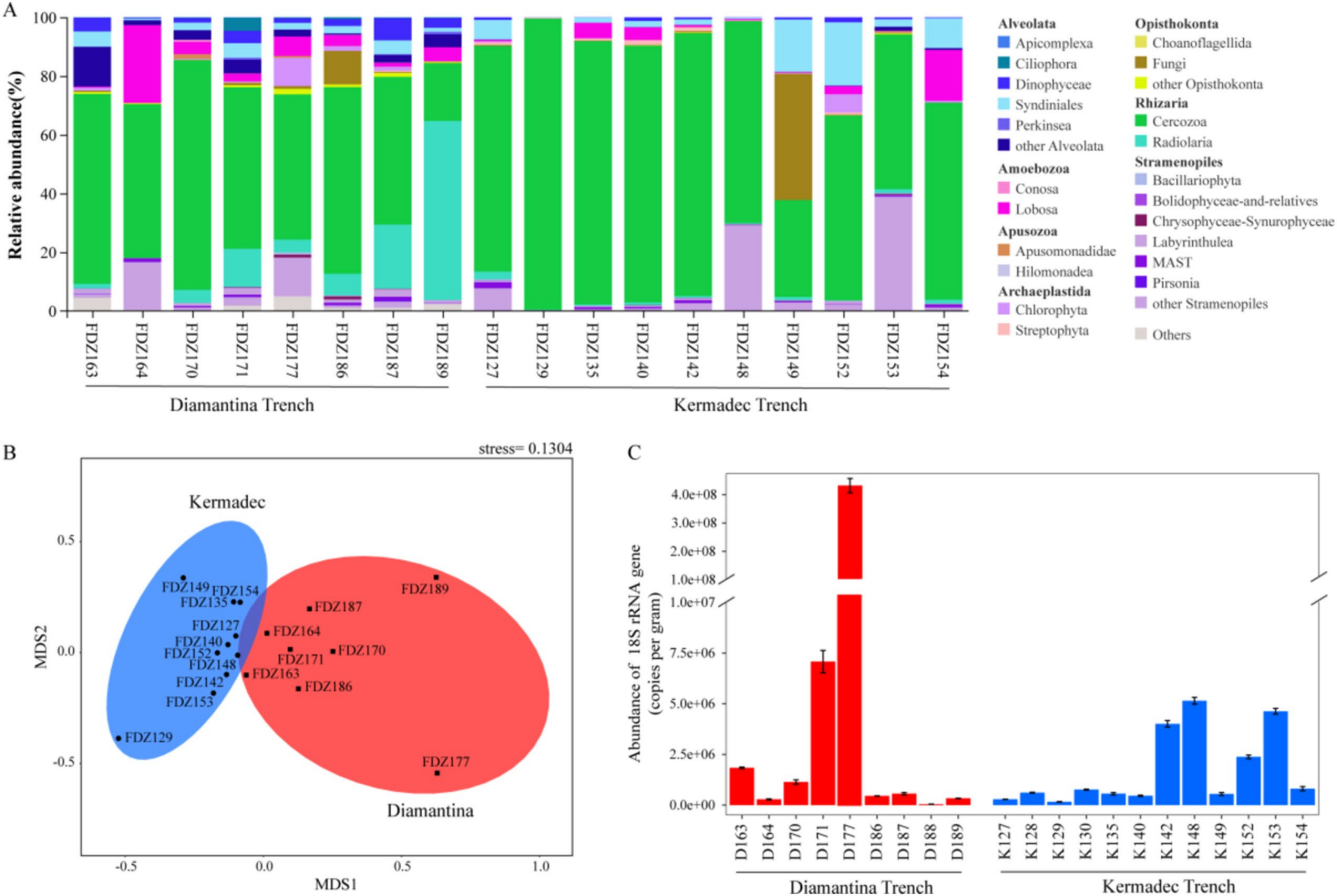
Community structure (A), NMDS plot (B), and gene copy number of 18S rRNA (C) of micro-eukaryote in the sediment of the Diamantina and Kermadec trenches. Error bars represent standard deviation.

For micro-eukaryotic sequences, members of super-group SAR (that is, Stramenopiles, Alveolata, and Rhizaria) were dominant across all sampling sites (Figure 3A). Other super groups (such as Apusozoa and Archaeplastida) altogether generally contributed less than 10% to the micro-eukaryotic sequences from each sample. In Alveolata assemblages, Dinophyceae and Syndiniales were the predominant groups, Cercozoa and Radiolaria were the dominant Rhizaria groups, and Labyrinthulea was the main component of the Stramenopile group. Among them, Cercozoa accounted for an obviously higher proportion at all stations. NMDS analysis showed that micro-eukaryotic communities of the two trenches were significantly different (ANOSIM, *p* < 0.05) (Figure 3B). Similar to bacterial communities, significantly lower gene copy numbers of micro-eukaryotic 18S rRNA genes were detected in the Kermadec Trench (ANOSIM, *p* < 0.05) (Figure 3C).

### 3.3 Comparison of the microbial communities

**Figure 4:**
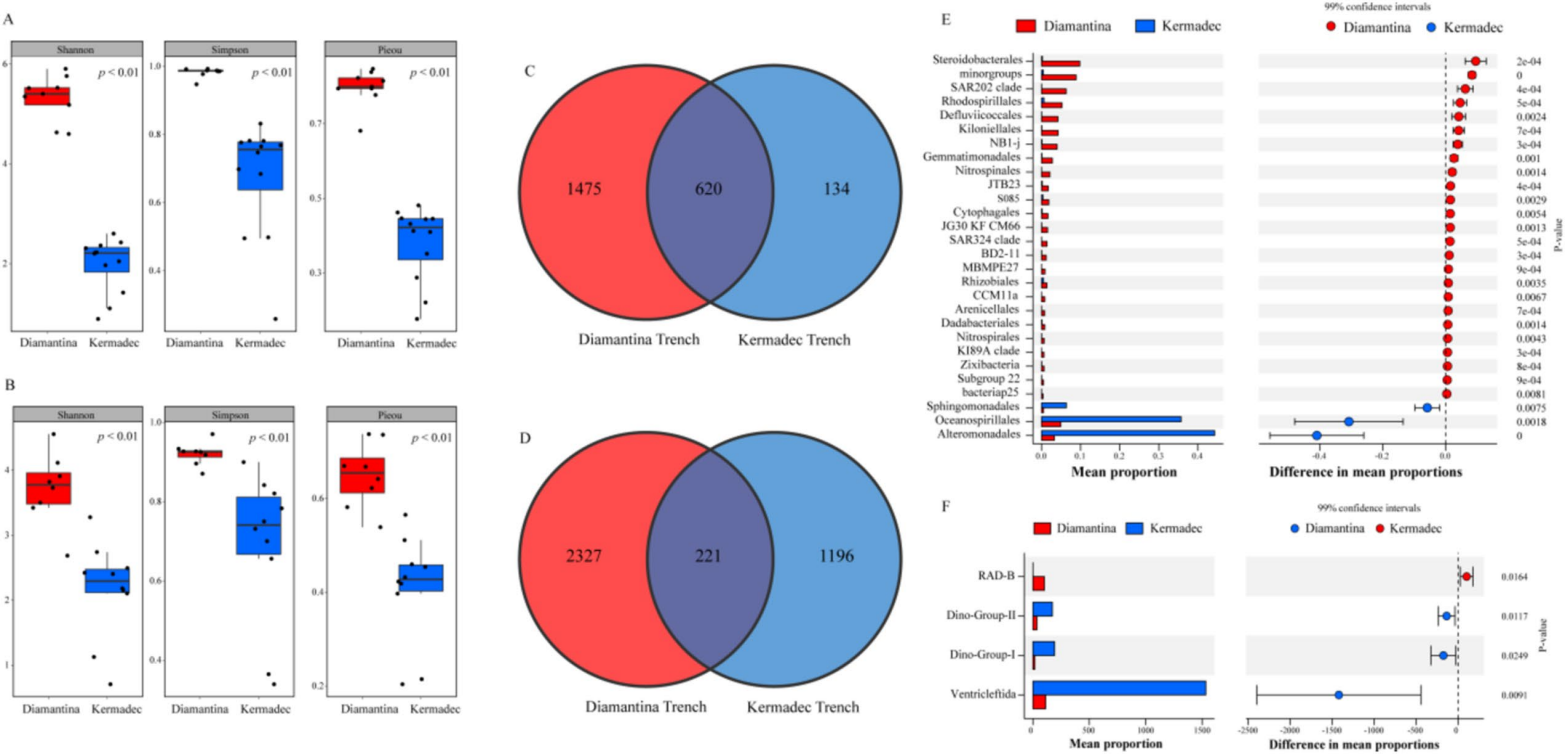
Community diversity index, Venn diagram, and extended error plots of bacteria (A,C,E) and micro-eukaryotic (B,D,F) communities between the Diamantina and Kermadec trenches. Extended error plots are presented in STAMP using SEED subsystems. The mean proportions in different categories are displayed in the bar graph. The colored circles (red and blue) indicate the 99% confidence intervals calculated using Welch’s t-test.

For bacterial and micro-eukaryotic communities, more specific/endemic ASVs existed in the Diamantina trenches than in the Kermadec trenches (Figures 4A,B), and 628 bacterial ASVs (Figure 4A) and 211 micro-eukaryotic ASVs (Figure 4B) were shared by both two trenches. In addition, the highest Shannon diversity index for bacterial (Figure 4C) and micro-eukaryotic (Figure 4D) communities was observed in the Diamantina Trench. Based on the STAMP analysis, significant bacterial ASV differences between the two trenches were found among the Steroidobacterales and SAR202 clade (more abundant in the Diamantina Trench), and the Alteromonadales and Oceanospirillales (more abundant in the Kermadec Trench) (Figure 4E). Micro-eukaryotic ASV differences were mostly found in the SAD-B and Ventricleftida (Figure 4F).

### 3.4 Assembly process of microbial communities

**Figure 5:**
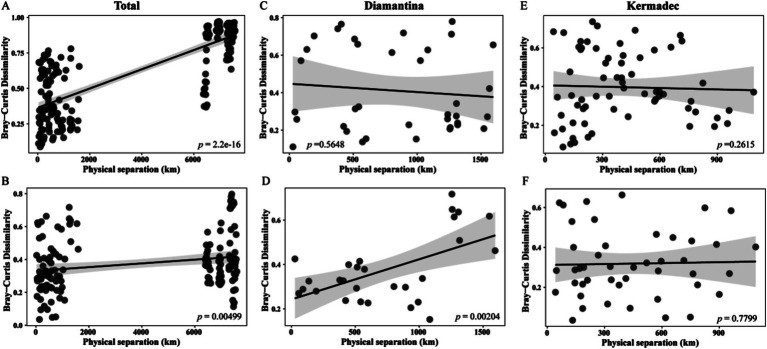
Co-relations between bacterial and micro-eukaryotic community dissimilarities and geographic distances for sampling sites in both trenches (A,B) and within the Diamantina (C,D) and Kermadec trenches (E,F).

**Figure 6:**
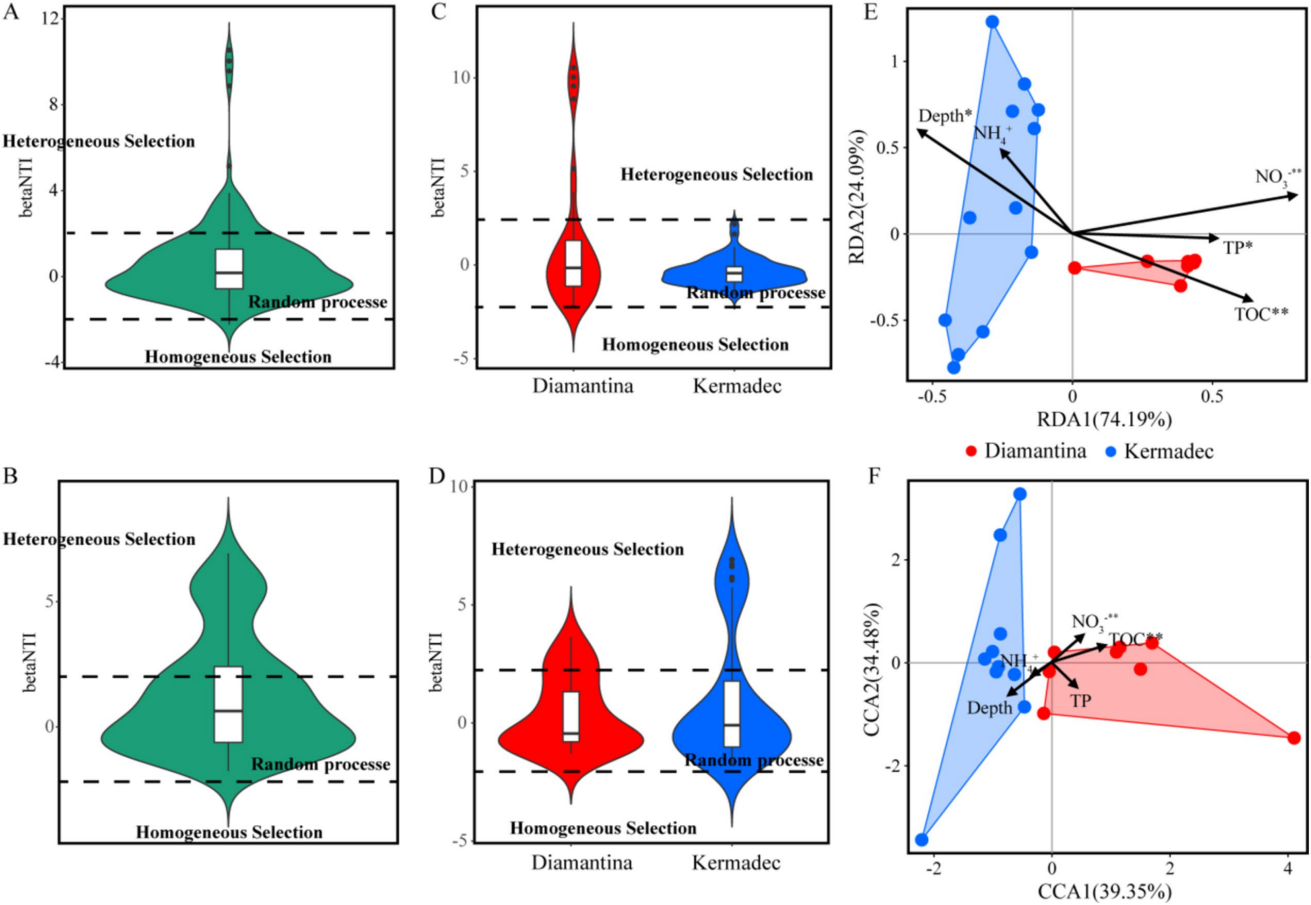
Null model and RDA/CCA with environmental variables of bacteria (A,C,E) and micro-eukaryotic (B,D,F) communities. Phylogenetic turnover (betaNTI) was calculated using a null model. Different ecological processes were represented by values of betaNTI with betaNTI > 2 indicating heterogeneous selection, |betaNTI| < 2 indicating random processes, and betaNTI < −2 indicating homogeneous selection.

The composition dissimilarity of integrated bacterial (Figure 5A) and micro-eukaryotic (Figure 5B) communities in both trenches significantly increased with geographical distance according to the distance–decay module (linear regression; *p* < 0.01). Within a single trench (Figures 5C–F), the micro-eukaryotic community in the Kermadec Trench showed a significant distance–decay pattern. A null model was applied to explore the relative importance of deterministic and stochastic processes in microbial community assembly. Random processes explained approximately 84.76 and 70.59% of the bacterial (Figure 6A) and micro-eukaryotic (Figure 6B) communities, respectively. Despite the dominance of random processes, deterministic (i.e., heterogeneous and homogeneous selection) processes exerted a stronger effect on the bacterial community (Figure 6C) of the Diamantina Trench and micro-eukaryotic community (Figure 6D) of the Kermadec Trench, respectively. After eliminating parameters with a variance inflation factor (VIF) > 10, five environmental factors were selected for further CCA/RDA analysis. The results showed that depth, TP, NO_3_^−^, and TOC had a significant impact on bacterial communities (Figure 6E), while depth, TOC, and NO_3_^−^ significantly influenced micro-eukaryotic communities (Figure 6F).

**Figure 7:**
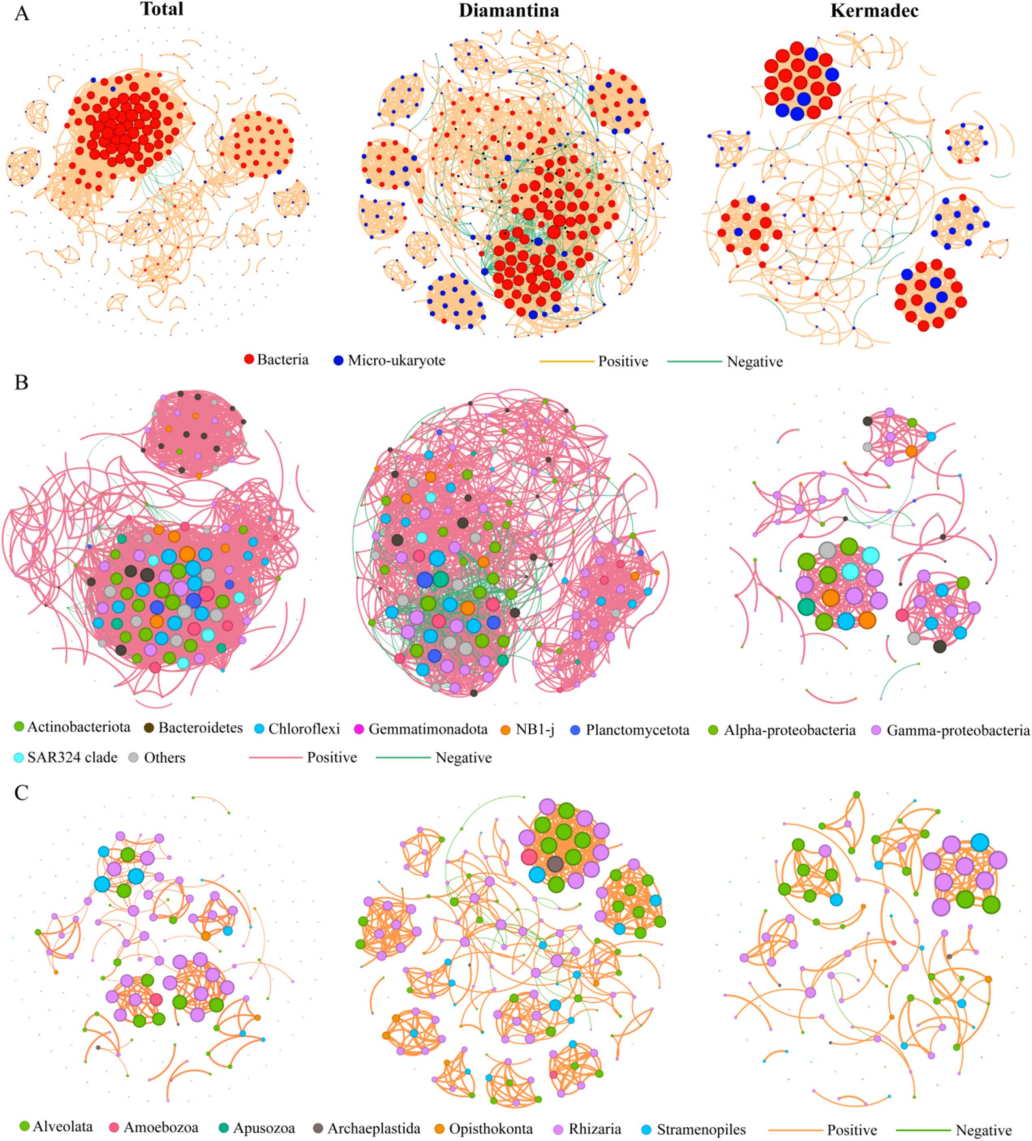
Co-occurrence networks for two domains (A), bacteria (B) and micro-eukaryote (C) for sampling sites in both trenches (total) and respective of the Diamantina and Kermadec trenches. A connection represents a strong (Spearman’s *r* > 0.8 or *r* < −0.8 and significant [*p* < 0.05]) correlation; that is, the thicker edges represent stronger correlations. The colored nodes represent bacterial and micro-eukaryotic groups. The size of each node is proportional to the number of connections it has (i.e., degree).

To elucidate the inter-domain interactions of bacterial and micro-eukaryotic ASVs, network analysis was performed with a total of 400 nodes and 3,181 links. Among these links in the network, 99.5% were positively correlated, indicating a positive interaction between bacterial and micro-eukaryotic ASVs dominated in the studied area (Figure 7A). Compared to the Kermadec Trench, the Diamantina Trench showed higher modularity, degree, betweenness, closeness, and eigenvector centrality, indicating higher network complexity for bacterial (Figure 7B) and micro-eukaryotic (Figure 7C) communities.

### 3.5 Potential functions and trophic status of microbes

Potential functions associated with carbon, nitrogen, and sulfur cycles for bacteria were inferred based on the 16S rRNA gene sequences using Tax4Fun (Supplementary Figure S1). Cellulose degradation (carbon cycle); nitrogen fixation, nitrification, and aerobic nitrite oxidation (nitrogen cycle); and thiosulfate respiration (sulfur cycle) were inferred as the major functional categories in the Diamantina Trench, whereas hydrocarbon degradation, denitrification, and sulfite oxidation were inferred to be more important in the Kermadec Trench. The types of carbohydrate degradation in the Kermadec Trench axis varied from northeast to southwest, and the abundance of genes related to sulfur oxidation was significantly higher than that in the Diamantina Trench (*p* < 0.05). For the potential trophic status of the micro-eukaryotic community, heterotrophic (e.g., Dinophyceae) and parasitic (e.g., Perkinsea and Syndiniales) types were predominant throughout the sediment samples (Supplementary Figure S2). Significantly higher heterotrophic and parasitic proportions were found in the Diamantina Trench (ANOSIM, *p* < 0.05).

## 4 Discussion

### 4.1 Distribution patterns of bacteria and micro-eukaryotes

Significantly different microbial diversity, sequence abundance, and microbial communities in the sediments between the two trenches were revealed, in agreement with the substantial differences in microbial community structures between the Mariana and Mussau Trenches reported previously (Liu et al., 2020). Although the Diamantina and Kermadec trenches are at the same latitude, they were located in different oceans and formed by different geological processes, which might support different microbiomes. These differences were likely attributed to the geographic separation, as well as the differences in tectonic activity and geochemical regimes (Jamieson, 2015; Peoples et al., 2019). The significantly lower sequence abundance and biodiversity of bacteria and micro-eukaryotes in the Kermadec Trench (which is deeper than the Diamantina Trench) may have resulted from the limited availability of labile nutrients after long-term burial at the trench’s bottom (Fu et al., 2020). This pattern has also been observed in the shallow seamounts and the Challenger Deep of the Mariana Trench (Zhang et al., 2023; Zhang and Jing, 2024).

The predominant heterotrophic bacterial lineages and their spatial distribution were also varied in each trench. Members of the Alteromonadales and Oceanospirillales, which are more dominant in the Kermadec Trench, have the capability of producing exopolysaccharides (Le Costaouëc et al., 2012; Gutierrez et al., 2013) that can trap nutrients and retain extracellular enzymes (Gutierrez et al., 2013). This capability might reflect an adaptation strategy to the low concentration of organic matter in the Kermadec Trench. Similarly, the hadal bacterial community consisted mostly of Alteromonadales and Oceanospirillales in the Mariana Trench (Wang et al., 2022). In contrast, more heterotrophic groups with low proportions each were detected in the Diamantina Trench, compared to the Kermadec and Mariana trenches (Wang et al., 2022). Those diverse heterotrophs discovered had the potential capability to degrade a broad variety of organic carbon and sulfur compounds, including recalcitrant molecules (Varela et al., 2008; Wasmund et al., 2016). Mutually cooperative strategies for accessing organic matter (Teske et al., 2011) may allow these complex heterotrophic groups to degrade oceanic organic matter in the Diamantina Trench.

For micro-eukaryotes, Radiolaria (mainly Cercozoa) occurred more frequently in the Kermadec Trench, consistent with their dominance in the Mariana Trench (Jing et al., 2018). It is widely recognized that Radiolaria are major contributors that transport organic carbon to the deep sea (Schnetzer et al., 2011), and they also form symbiotic associations among many other living microbes (e.g., dinoflagellates, Prasinophyceae, and Prymnesiophyceae) (Decelle et al., 2012). The dominance of Radiolaria recorded in the Kermadec Trench, especially in the middle of the axis, might indicate that they have a vital role in sustaining the stability of the microbial ecosystems in the Kermadec Trench. Compared to the Kermadec Trench, higher micro-eukaryotic diversity, including members of the Dinophyceae, was discovered in the Diamantina Trench. Interestingly, Dinophyceae were present from the surface to the hadal zone in the Mariana Trench, which could be attributed to sinking cysts. This might the main reason for the presence of photoautotrophic cells in the dark ocean (Xu et al., 2018). In addition, a mixotrophic lifestyle could also be a potential reason for those active photosynthetic groups found in the Mariana Trench (Jing et al., 2018).

### 4.2 Impact of stochastic processes on the microbial community assembly

The relative importance of microbial deterministic and stochastic processes varied with different trenches, a possible consequence of heterogeneous habitat features and seawater-driven dispersal limitation (Li et al., 2023). Stochastic and deterministic processes governed the micro-eukaryotic community assembly in cold seeps (Xu et al., 2023) and in trench sediments (Zhang et al., 2023), respectively. In the Mariana Trench, macrofaunal (Giguère and Tunnicliffe, 2021) and prokaryotic (Li et al., 2024) community assembly was mainly affected by deterministic and stochastic processes, respectively. In the current study, stochastic processes (e.g., dispersal limitation and drift) were significant drivers influencing bacterial and micro-eukaryotic assembly and biogeography, consistent with the prokaryotic community assembly processes of the Yap Trench sediments (Sun et al., 2024) and vertical water column in the Mariana Trench (Li et al., 2024). The weak impact of competition on microbial population structure in the trenches (see Figure 7) could enhance stochasticity and obscure linkages between microbial community structure and environmental variables (Evans et al., 2017). The stochastic processes (mainly dispersal limitation) impacted bacteria to a greater degree than micro-eukaryotes. In contrast, only stochastic processes influenced the community distributions of all three domains (i.e., archaea, bacteria, and micro-eukaryotes) in the Mariana Trench (Li et al., 2024). It was already known that the dispersal limitation/drift exhibited distinct effects on the shift and assembly of prokaryotic and eukaryotic metacommunities (Kirchman, 2016), due to differences in their population size, proliferation, and death rates (Stegen et al., 2013). For example, bacteria and archaea generally possess larger population sizes than micro-eukaryotes and are thus less affected by drifting (Wu et al., 2018), which could potentially influence the strength of dispersal limitation that operates alongside drift (Stegen et al., 2013). In addition, the distinctions in selective grazing and trophic levels among prey, degrader (bacteria), and predator (micro-eukaryote) might also influence the relative contribution of stochastic processes on multi-domain microbiomes (Livingston et al., 2017).

In the current study, environmental selection also governed communities in surficial sediment. Although stochastic processes prevail in the assembly of microbes, a higher proportion of heterogeneous selection of deterministic processes was found in the bacterial community of the Diamantina Trench and the micro-eukaryotic community of the Kermadec Trench. This suggests that changes in environmental conditions might lead to community divergence through the mediation of heterogeneous selection (Zhang et al., 2020). Unlike the findings of this study, a homogeneous selection, which is part of deterministic processes, controlled the prokaryotic community assembly in the hadal sediments of the Yap Trench as sediment depth increased (Sun et al., 2024). Although TOC and NO_3_^−^ significantly affected both microbial domains, depth and TP also had significant impacts on bacterial communities rather than micro-eukaryotes, indicating that different domains had distinct responses to environmental variables in the trench. Consistent with previous research, micro-eukaryotic communities are more susceptible to environmental selection and species sorting compared to bacterial communities since they possess a more limited tendency to enter dormancy (Massana and Logares, 2013).

### 4.3 Stability and complexity of the microbial community

Microbial interactions have been proposed as biotic drivers that influence the composition of microbial communities (Calcagno et al., 2017). The high modularity observed in the Diamantina (0.936) and Kermadec (0.823) trenches indicates that microbial interactions and cross-module associations among taxa are probably widespread in the trench ecosystem. Plentiful microbial groups with both positive and negative relations were clustered together, suggesting that microbial associations in the two trenches were diverse and complex, although the complexity of the microbial network differed between the two trenches. According to MacArthur’s argument (MacArthur, 1955), the complexity of ecosystems contributes to their stability. Our results demonstrate that the environmental heterogeneity did not lead to the destabilization of microbiome communities after long-term adaptation and coevolution in the trench. The complexity of associations of multi-domain microbiomes has also been reported to make a significant contribution to community stability in the Mariana Trench (Li et al., 2024). In addition, the various combinations of species and relationships performed by different major groups and taxa in these two trenches might stand for specific adaptation strategies to unique habitats. Heterotrophy and parasitism were common survival strategies in the two studied trenches for micro-eukaryotic communities. Parasitism can modify the composition and dynamics of food webs by affecting host characteristics and abundance (Frainer et al., 2018). Its important role has also been reported in extreme environments, such as seamounts, cold seeps, and hydrothermal vents (Zhang et al., 2021, 2023).

The annotated function could infer an assessment of potential microbial metabolic activities (Steinert et al., 2019). This potential difference in types of carbohydrate degradation for bacterial communities in the Kermadec Trench axis varied from northeast to southwest, which might be caused by a strong heterogeneity in depositional organic matter characteristics in the trench (Xu et al., 2021). The co-occurrence networks of cross-domain microbiome associations between the two trenches exhibited more commonalities with similar complexity and the ratio of positive links. However, different major groups and taxa performed various combinations of relationships, which might represent specific strategies for adaptation to distinct habitats. The varied contribution of key taxa from different domains to community integrity confirmed the inference of niche and functional redundancy, where different taxa possibly share the same ecological functions in the trench ecosystem. In addition, different bacterial communities at sampling sites between two trenches performed similar ecosystem functions in our study, uncovering the presence of functional redundancy patterns. Functional redundancy was prevalent in trench ecosystems (Liu et al., 2022; Sun et al., 2024), where some taxa that shared similar functions were more inclined to ecological drift (Zhou and Ning, 2017). This is consistent with our results of the null model, which showed that drift plays a more important role in the ecological processes of the trench ecosystem. However, those potential functions were predicted at the DNA level; further validation at the transcriptome and metabolome levels would be needed in the future.

## Data Availability

The datasets presented in this study can be found in online repositories. The name of the repository and accession number can be found at: https://www.ncbi.nlm.nih.gov/, PRJNA1062464.

## Footnotes

^1^ www.novogene.com
